# Bacterial Proteomics and Antibiotic Resistance Identification: Is Single-Cell Analysis a Worthwhile Pursuit?

**DOI:** 10.3390/pathogens14111127

**Published:** 2025-11-05

**Authors:** Navid J. Ayon

**Affiliations:** Analytical Development Laboratory, United States Pharmacopeia, Rockville, MD 20852, USA; navid.ayon@usp.org

**Keywords:** antimicrobial resistance, antibiotic susceptibility, single-cell proteomics, bottom-up proteomics, top-down proteomics, drag safety, antimicrobial therapy, infectious disease, pathogen, antibiotic stewardship, public health

## Abstract

Antibiotic resistance is a major threat to global public health. It is vital to understand the mechanism of antibiotic resistance development to prevent the emergence of new pan-resistant pathogenic bacteria and to develop new antibiotics. Measuring the differences in proteins among single bacterial cells can aid in identifying antibiotic resistance and antibiotic susceptibility due to their regulatory roles in bacterial physiology and homeostasis. Although single-cell proteomics has been successful in mammalian systems, attaining comparable performance in bacteria remains challenging due to the extremely limited proteome content of a single bacterial cell. This review discusses the role of proteomics analysis in determining antibiotic resistance and the various mass spectrometry-based strategies that have been successful in detecting protein biomarkers for antibiotic resistance from bulk proteomics analysis. It highlights the core challenges of bacterial single-cell proteomics in contrast to mammalian systems, explores emerging technologies, and the proteomes beyond the cells in studying antibiotic resistance development and antibiotic susceptibility testing.

## 1. Introduction

Proteins are one of the major constituents of any biological system that are virtually involved in all cellular processes [1]. All proteins expressed by an organism are collectively called the proteome, and their study is known as proteomics [2]. Each organism has its distinct proteome; in fact, different organs, tissues, or even cells within an organism have their own unique proteomes, differences in which are now possible to study due to the advancements made in the field of proteomics. Unlike the genome, the proteome is a dynamic biological layer that differs spatially and over time [3]. Proteins are also closer to biological functions than genes or mRNA [4]. Hence, the ability to capture spatial or time-resolved snapshots of proteins within an organism could help unlock new insights into its biological processes in real time. Proteins are large biomolecules composed of polypeptide chains made from proteinogenic amino acids and their stereoisomers [5,6,7], which carry instructions from the genome through the transcriptome to regulate various functions effected by the metabolites [8]. Besides reflecting abnormalities inherited from the genome or transcriptome, proteins can exhibit post-translational abnormalities that are not detected in genomic or transcriptomic analyses. Variants of a protein that stem from a single gene but differ from the canonical form due to genetic variations, mutations, alternative splicing of mRNA, or post-translational modifications (PTMs) are known as proteoforms [9], which can be hallmarks of pathological conditions. Proteomics analysis enables the measurement of protein quantity, modifications, structures, localization, aggregation behavior, and interactions with other molecules, which holds a wealth of information for the science of health and disease. Mass spectrometry (MS) is a versatile technology that has enabled measuring the proteome [9] and demonstrated to yield information about PTMs, stoichiometry, and native structure of proteins at attomolar range (1 target protein per 10^18^ molecules) [10].

Multicellular organisms contain different types of cells carrying out various biological functions that are necessary for their growth, development, operation, and survival [11]. Differences in cell population in each cell type exist, even in a homogenous tissue [12]. Cells of different types work concertedly to perform these functions, which also involve intercellular communication maintained by proteins and their interactions with other molecules. Most cell-based protein assays involve pooling several cells to yield enough material, i.e., the proteome needed to meet the sensitivity threshold of the analytical technology, which does not preserve the intrinsic heterogeneity of the cells [13]. Identifying cell subpopulation and heterogeneity by its proteomic composition has been challenging until the emergence of single-cell proteomics (SCP), which enabled the identification of cellular diversity, heterogeneity, and subcellular organization based on proteomic differences in single cells [14]. Single Cell ProtEomics by Mass Spectrometry (SCoPE-MS) presented unprecedented analytical ability to measure proteins from individual human cells using carrier proteome multiplexed with isobaric tandem mass tag (TMT), and researchers around the globe are continuously pursuing new technologies aiming to achieve more sensitivity and proteome coverage to measure smaller single cells [15]. Analyzing proteoforms from a single-cell-derived proteome was not possible until recently, when Su and Kelleher developed a nanospray desorption electrospray ionization (nano-DESI) single-cell Proteoform imaging Mass Spectrometry (scPiMS) technique that extracts and detects proteins directly from single mouse embryonic fibroblast (NIH 3T3) cells at a raw rate of <10 s/cell [16]. Although they could detect hundreds of proteoforms, only 41 were identified, all from 38 abundant proteins. They further utilized this workflow to explore the proteoform landscape of 10,809 single cells dissociated from rat brain hippocampus and assigned >93% of the single cells into astrocytes, microglia, and neurons based on their proteoform signatures at a throughput of ~1000 cells/day [17]; an unprecedented achievement for single-cell proteoform analysis.

Nucleic acid-based approaches, such as genomics and transcriptomics analyses, can identify differences between cells based on their genetic composition and infer protein expression levels. However, these techniques cannot provide information on any PTMs, which are often the indicators of critical pathological conditions. Next-generation sequencing (NGS) technology can address the sensitivity issues caused by low abundance by amplifying the target nucleic acid using the polymerase chain reaction (PCR) at the single cell level [18,19]. Such a multiplication technique is still not available for proteins [20]; hence, SCP depends on high-performing sample cleanup workflows, ultrasensitive mass spectrometry instrumentation, and innovative data acquisition techniques to achieve greater sensitivity and has enabled quantification of more than 1000 proteins from a single mammalian cell [21]. SCP could provide information on PTMs, single point mutation, more specifically single nucleotide polymorphism (SNP), ligand binding, protein interactions, and signaling between cells, among other important biological events that are crucial to understanding disease biology. Besides advanced sample preparation, hardware configurations, and acquisition techniques [22,23], the success of SCP is heavily dependent on the size of the single cells, the ability to sort them, and a sufficient proteome for data acquisition.

Antibacterial susceptibility testing checks and identifies the antibiotics that are effective against a bacterium, for which knowing its identity is essential. There are several bacterial typing methods, such as serotyping, biotyping, sequence typing, etc., and identification of bacteria based on their protein profiles is known as proteotyping, which has higher discriminatory capability to distinguish closely associated bacterial strains [24]. Mass spectrometry (MS)-based proteomics has revolutionized bacterial identification and classification, and enabled distinguishing bacteria at the genus, species, and sometimes at the subspecies level [25,26,27] at a faster speed, in less than 2 min [28], making it a valuable tool for clinical diagnostics. MS-based proteomics also enabled studying molecular and biochemical mechanisms of bacterial growth and antibiotic action, disease-causing ability, i.e., virulence, drug resistance development, and drug target identification [5,29,30,31,32]. Bottom-up proteomics has demonstrated its utility in identifying protein biomarkers for antibiotic resistance [26,33,34]. Thus, proteomics could potentially be used as a complementary approach to genomics and transcriptomics to identify resistant bacterial strains. This work reviews how proteomics analysis enables the identification of antibiotic resistance, with examples from top-down and bottom-up proteomics studies. It critically examines the primary challenges in bacterial single-cell proteomics and explores the emerging technologies that could potentially facilitate future endeavors in the field of bacterial SCP. It then discusses proteomes beyond the cell, such as secretory, membrane, and biofilm proteomes, which may provide critical insights into the development of antibiotic resistance. Finally, this review assesses the potential and the feasibility of bacterial SCP for routine clinical microbiology applications.

## 2. Antibiotic Resistance and Its Identification

**Figure 1:**
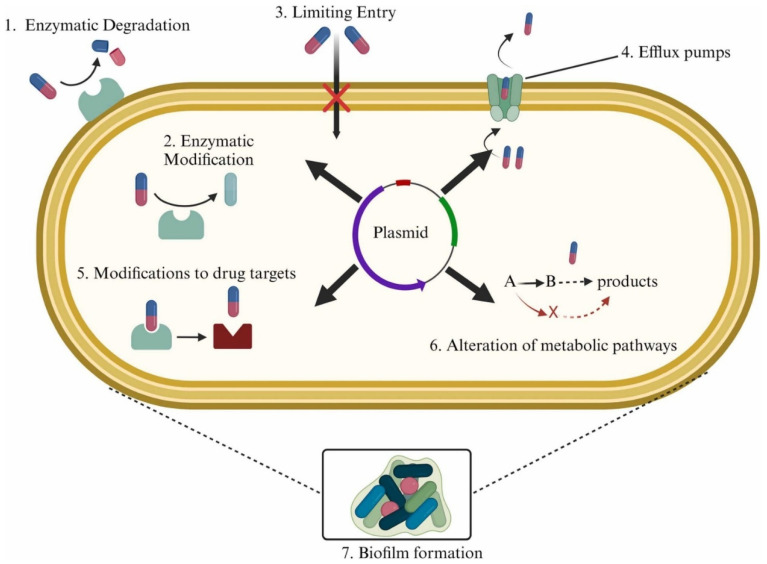
Mechanisms of antibiotic resistance development in bacteria [37].

Antibiotic resistance is the ability of bacteria to withstand exposure to antibiotics to which they were previously susceptible [35]. Bacteria develop resistance through various mechanisms, including genetic, biochemical, or phenotypic adaptations that could either modify the target protein and reduce its interaction with antibiotics, or express proteins that degrade the antibiotics or pump them out of the cell before they reach therapeutic concentrations [36]. Additionally, bacteria can adopt alternative metabolic pathways to bypass the antibiotics’ action [37] or form biofilms to limit their penetration into cells, creating tolerant zones, and promoting genetic changes [38] that lead to the development of antibiotic resistance. Common mechanisms of antibiotic resistance development are summarized in Figure 1 [37]. With any of these events in place, existing bacteria adapt to adverse conditions, and the new strains growing inside the host develop mutations, making them resistant to the current antibiotic regimen. Inappropriate use of antibiotics can lead to the emergence of antibiotic-resistant strains in one patient, which can then spread to other patients who do not use antibiotics, underscoring the severity of this public health concern [38].

Primarily, bacteria acquire resistance to a particular antibiotic by developing a new mutation or acquiring it from another bacterium through the transfer of genetic materials, e.g., DNA. Bacteria often acquire resistance through cohabitation with other bacteria, a common phenomenon in bacterial communities [39]. Overuse or misuse of antibiotics, inappropriate prescribing, and lack of stewardship from both physicians and patients are the major factors contributing to the development of antibiotic resistance, and the situation is exacerbated by the scarcity of new or new classes of antibiotics [40,41]. The direct result of increased antibiotic resistance is increased morbidity and mortality due to a lack of effective therapy. Approximately 700,000 people lose their lives globally due to drug-resistant infections each year [42]. In the United States alone, antibiotic resistance takes approximately 23,000 lives every year and costs over $20 billion in additional medical expenses [43], whereas in Europe, the annual mortality rate is 25,000 patients a year [42]. It is also estimated that nearly 10 million people are predicted to lose their lives across the world by the year 2050 if new lines of therapy are not available, and this will add $300 billion to $1 trillion to the healthcare cost caused by intensive care unit (ICU) usage, isolation, and long-term hospitalization [44]. Antibiotic resistance also has secondary healthcare repercussions, including challenges during organ transplantation, where the recipient is vulnerable to infections [45], and during chemotherapy treatment for cancer patients, where antibiotic resistance impedes physicians from administering antibiotics to treat infections [43,46]. There are other serious economic consequences affecting gross domestic product (GDP) and global trade, with a long-term burden for both developed and underdeveloped countries [42]. Resistance to multiple antibiotics, known as multidrug resistance, has been reported in various bacterial species, including commensal flora and common pathogenic strains such as *Escherichia coli*, *Campylobacter* spp., *Enterococcus* spp., *Salmonella* spp., *Listeria* spp., and *Staphylococcus* spp. indicating the ease of transmission of drug resistance genes between various bacterial species and animals and humans [47]. Hence, it is clear that antibiotic resistance is a pressing issue and a serious public health concern, and addressing it starts with rapid and accurate identification of resistance.

**Figure 2:**
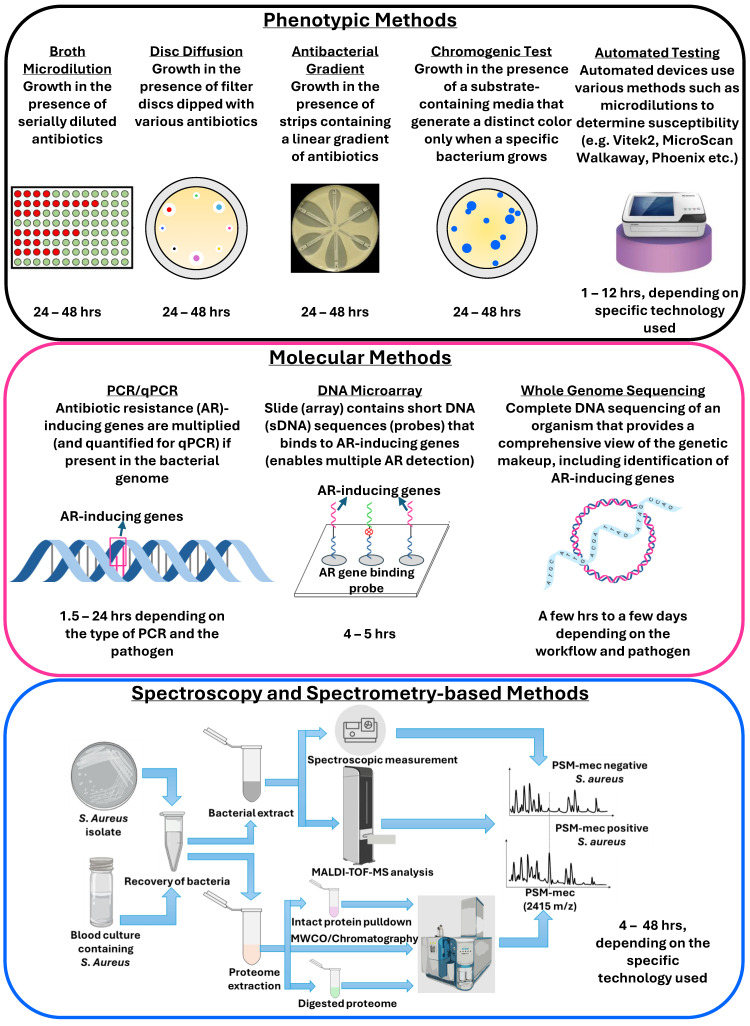
Methods for antibiotic resistance and susceptibility testing.

The most commonly used methods for antibiotic resistance detection are based on phenotypic and molecular characterization, with newer techniques such as those that are based on spectroscopy and spectrometry (Figure 2) [45].

Numerous excellent reviews are available discussing technologies for antibiotic susceptibility testing and resistance detection, covering both routinely used methods and advanced workflows [45,48,49]. Briefly, phenotypic methods such as disc diffusion, minimum inhibitory concentration (broth and agar dilution), gradient, chromogenic, and colorimetric tests rely on tens of thousands to millions of bacterial cells, require 20–48 h, and different antibiotics’ samples to test susceptibility. Several automated and semi-automated devices for microdilution-based susceptibility testing, such as the VITEK 2 (bioMérieux, Marcy-l’Étoile, France), MicroScan Walkaway (Dade-Behring MicroScan, Deerfield, IL, USA), and Phoenix system (BD Diagnostic Systems, Baltimore, MD, USA), have been introduced in the market that can produce results within 6–12 h [50]. Genotypic methods, such as PCR, isothermal amplification, and DNA microarrays, detect differences in genetic composition by measuring DNA or mRNA and mutations that cause antibiotic resistance, and are generally faster than phenotypic methods, providing results in hours [51]. However, such approaches only detect known resistance-causing genes and cannot identify the novel ones. Whole genome sequencing (WGS), on the other hand, can detect all genes involved in the development of resistance by comparing it to a control bacterial strain. Besides genomics, transcriptomics analysis also demonstrated success in resistance identification. Although WGS and related technologies correlate highly with phenotypic methods [52], they are not routinely used in clinical testing due to the high cost and turnaround time [53].

Matrix-assisted laser desorption/ionization-time-of-flight mass spectrometry (MALDI-TOF-MS) has demonstrated success in identifying bacterial species and antibiotic resistance by analyzing protein biomarkers, relative growth rates, and stable isotope uptake in resistant bacteria upon antibiotic exposure [54,55]. However, most MALDI-based approaches depend on smaller proteins (<20 kDa), most of which are part of the structural proteome, such as ribosomal proteins, limiting strain-level differentiation [56]. Resistance-inducing proteins, such as proteins with enzymatic activity, are generally less abundant in living cells compared to structural proteins and can have a reduced expression level until exposed to antibiotics, as well as can also be embedded in the membrane compared to being freely available in the cytoplasm, making it difficult to analyze them via MALDI-TOF-MS-based workflows [57]. Spectroscopy techniques such as atomic force microscopy, infrared, and Raman spectroscopy have also demonstrated success in detecting antibiotic resistance [48]. However, none of these techniques can detect protein biomarkers in individual cells. Hence, faster methods are required to detect antibiotic resistance and determine antibiotic susceptibility in clinical specimens, thereby helping clinicians select the right antibiotics for treating bacterial infections.

## 3. Bacterial Proteomics and Antibiotic Resistance Identification

Success in treating a bacterial infection depends on selecting the right antibiotic, which requires identifying the bacterial species, their antibiotic susceptibility, and their resistance profile. Although most routinely used workflows for resistance detection are based on genes and/or transcripts, resistance identification at the protein level should be a better indicator of antibiotic resistance, as resistance development is almost exclusively mediated by proteins [58,59]. Liquid chromatography tandem mass spectrometry (LC-MS/MS)-based targeted proteomics, which relies on the selective monitoring of resistance-inducing peptides, has demonstrated success in identifying resistance determinants in samples with known species information [60,61,62,63,64,65], establishing that resistance determination based on protein can be performed. MALDI-TOF-MS-based workflows identify bacterial strains by analyzing protein fingerprints from whole-cell extracts and have demonstrated their utility in clinical settings [66]. The utility of MALDI and electrospray ionization (ESI)-based approaches to identify antibiotic resistance-inducing proteins has been reported since 2000, as can be seen in the study by Wilcox et al., where they identified protein biomarkers for antibiotic resistance against streptomycin, erythromycin, and spectinomycin in three strains of *E. coli* [67]. Bacterial proteomics analysis successfully identified many proteins responsible for inducing resistance to various classes of antibiotics, including beta-lactams, aminoglycosides, and fluoroquinolones, which are comprehensively reviewed by Sulaiman and Lam [68].

Mutations in genes or the acquisition of new resistance genes alter the structure and function of antibiotic-targeting proteins, thereby reducing the antibiotic’s therapeutic efficacy [36]. Recent studies reported that modification of proteins that are not the target of antibiotics or that inactivate antibiotics but are related to the central carbon or energy metabolism of bacterial cells can also lead to resistance development [69]. Physiological processes that help bacteria adapt to stressors, such as antibiotics, are regulated by proteins, and changes in these proteins can explain the mechanisms underlying cell damage or death [70]. Thus, studying the proteomes of antibiotic-sensitive and resistant bacteria and how they respond to antibiotics is expected to yield new insights into bacterial physiology, coping mechanisms, the mechanisms of action of antibiotics, and the development of resistance.

Bottom-up proteomics (BUP), where protein identification is based on genome sequences, holds potential for simultaneous species and resistance identification [71]. However, the single gene-to-single protein hypothesis is no longer valid [72], and a single gene can encode multiple protein variants, some of which are modified post-translationally and have different structures from the canonical forms [73]. As BUP involves digesting proteins into peptides for mass spectrometry analysis, it struggles to link identified modifications to intact proteins [74]. The best MS strategy for studying modified, non-canonical proteoforms is to measure them in their intact form, a technique known as top-down proteomics (TDP) [75]. TDP preserves intact protein structure and can be beneficial for identifying non-canonical versions of proteins, including post-translational modifications, single-point mutations, and splicing variants, and can help identify antibiotic resistance-inducing proteins or proteoforms, particularly if the non-canonical proteins (i.e., proteoforms) drive resistance development. In the following sections, identification of antibiotic resistance via MALDI and non-MALDI-based bottom-up and top-down proteomics is explored.

### 3.1. MALDI-TOF-MS-Based Top-Down Proteomics for Antibiotic Resistance Identification

**Figure 3:**
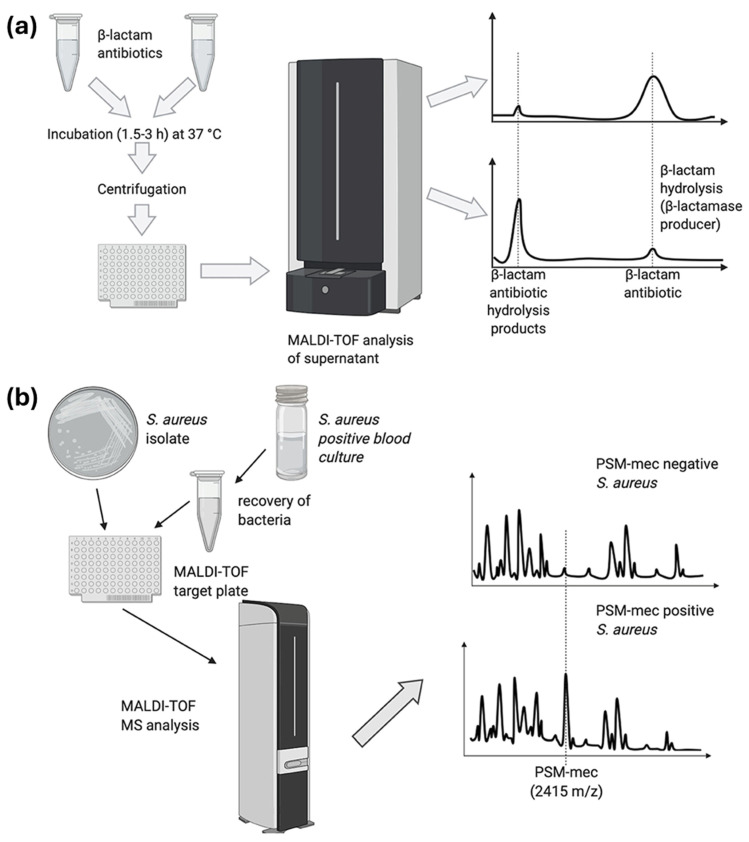
(**a**) Detection of β-lactamase producers by MALDI-TOF-MS based on the hydrolysis of the target β-lactam antibiotic, as visualized by peak disappearance. (**b**) Schematic representation of the MALDI-TOF-MS method used to discriminate *Staphylococcus aureus* strains based on the presence of the PSM-mec peak [54].

Based on the pioneering work of Anhalt and Fenselau, whole-cell mass spectrometry has revolutionized microbial identification using MALDI-TOF-MS as a gold standard for bacterial identification and phylogenetic classification [76,77]. In addition to microbial identification, MALDI-TOF-MS-based top-down proteomics approaches demonstrated success in identifying protein biomarkers for antibiotic resistance [78]. Besides soluble proteins, MALDI-TOF-MS helped in identifying single amino acid mutations in a membrane protein, mycobacterial ATP synthase c subunit, in less than 4 hrs, which is responsible for inducing resistance to bedaquiline [79]. MALDI-TOF-MS-based approaches have also been used to identify antibiotic resistance in bacteria by detecting antibiotics’ hydrolytic products, e.g., for beta-lactams (Figure 3a), as well as marker proteins inducing resistance, e.g., PSM-mec in *Staphylococcus aureus*, yielding resistance to methicillin (Figure 3b) [54].

Several excellent articles comprehensively reviewed the utility of MALDI-TOF MS in detecting biomarkers for antibacterial resistance [80,81,82]. Viboud et al. performed a systematic analysis of a MALDI-TOF-MS-based study and listed workflows detecting antibiotic resistance based on bacterial growth in the presence of antibiotics, as well as based on peaks representing drug resistance in whole-cell mass spectrometry data [83]. In addition, routine MALDI-TOF-MS-based proteotyping, integration with offline liquid chromatography [84], and Fourier-transform infrared (FTIR) spectroscopy [85] have demonstrated utility in exploring antibiotic resistance besides strain-level identification. In recent years, the importance of machine learning and artificial intelligence for MALDI-TOF-MS-based workflows in predicting antibiotic resistance in bacteria has been studied [86,87], and such integrations are adopted for a range of bacterial strains, e.g., Pseudomonas *aeruginosa* [88], *Staphylococcus epidermidis* [89], and *Escherichia coli* [90].

However, MALDI has lower ionization efficiency, resulting in lower sensitivity than ESI-based approaches, and predominantly yields singly charged ions [91], thereby leading to lower proteome coverage [92]. Due to these limitations, MALDI-TOF-MS-based workflows for single-cell proteomics analysis require careful consideration, particularly for extremely small single cells with a very small proteome. Several studies were reported in single-cell bacterial metabolomics, where small-molecule metabolites were detected in sub-2 pL to 1 nL volumes [93,94]. Reduced nutrient media has been used in a study by Schirmer et al. which showed accumulation of lysine in a 1 nL volume on a chip [95]. Electroporation-assisted intracellular metabolome extraction in a nanoESI emitter tip in ~1.5 pL demonstrated the detection of nucleotide, amino acids, and sugars from a single yeast (*Saccharomyces cerevisiae*) cell [93]. Xu et al. developed a high-throughput screening platform combining MALDI-MS with printed droplet microfluidics to characterize enzyme activity of type-3 polyketide synthase (PKS) from single cells in 300 pL droplets and screened a yeast mutant library, which could potentially be explored to identify mutation(s) conferring resistance to antibiotics [96]. Although single-cell analysis was performed for metabolomics in this study, this approach could, in principle, be applied to proteomics.

### 3.2. Non-MALDI-TOF-MS-Based Top-Down Proteomics for Antibiotic Resistance Identification

**Figure 4:**
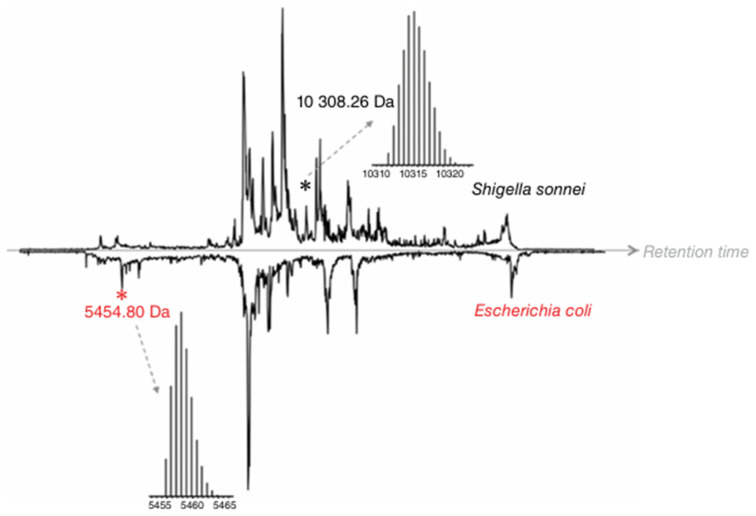
Comparison of the total-ion chromatograms obtained for the LC-MS analysis of intact proteins extracted from *S. sonnei* and *E. coli* lysates. Black and red asterisks indicate a unique protein peak for *S. sonnei* and *E. coli*, respectively [103]. © Wiley, reprinted with permission.

Top-down proteomics, in contrast to BUP, measures proteins in intact form without digestion and enables studying protein variants not directly encoded by the genome [97] but resulted from alternative splicing, allelic variation, in vivo proteolysis, or post-translational modification [98,99,100]. Modifications of expressed proteins due to any of the events mentioned above potentially can be reflective of a pathological condition, e.g., infection [101]. As per the proteoform hypothesis, proteoforms have the greatest ability to differentiate real biological differences in complex samples, and TDP is the best available technology to date for intact proteoform analysis [102]. MALDI-TOF-MS-based proteotyping uses intact proteins as markers for bacterial identification, which depends on the completeness of the database used for spectral matching of the measured data. Hence, without any front-end separation, it may not be able to identify or differentiate between bacterial species, as seen for *Escherichia coli* and *Shigella sonnei*, which have closely related MS profiles [103] and was successfully distinguished by LC-ESI-MS-based untargeted TDP analysis (Figure 4).

Compared to mammalian systems, bacteria exhibit fewer PTMs, making the proteoform analysis comparatively less complex [104]. Melo et al. reported the identification of 1125 proteoforms of 273 proteins from *Corynebacterium glutamicum* proteome, including several PTMs, such as acetylation, oxidation, and formylation of proteins related to amino acid production, protein secretion, and oxidative stress [99]. Additionally, bacterial proteins and their modifications are highly conserved across different species, especially within the same phylum [105]. These features make TDP an attractive technique for identifying proteomic differences, especially at the proteoform level among closely related strains.

There are significant differences between the proteins that are detected using MALDI- and ESI-based ionization in terms of their amino acid compositions, charged states, hydrophobicity, and modifications [106]. As discussed before, MALDI predominantly generates singly charged ions. Although this works for whole-cell mass spectrometry, it limits the ability to study high-molecular-weight proteins that generate multiply charged envelopes. Although ESI may suffer from ion suppression when samples are not free of salts and other matrix components, it offers higher ionization efficiency, is easy to integrate with chromatographic separation systems, and thus provides better sensitivity and specificity; hence, it is a better strategy for exploring intact proteins and proteoforms.

**Figure 5:**
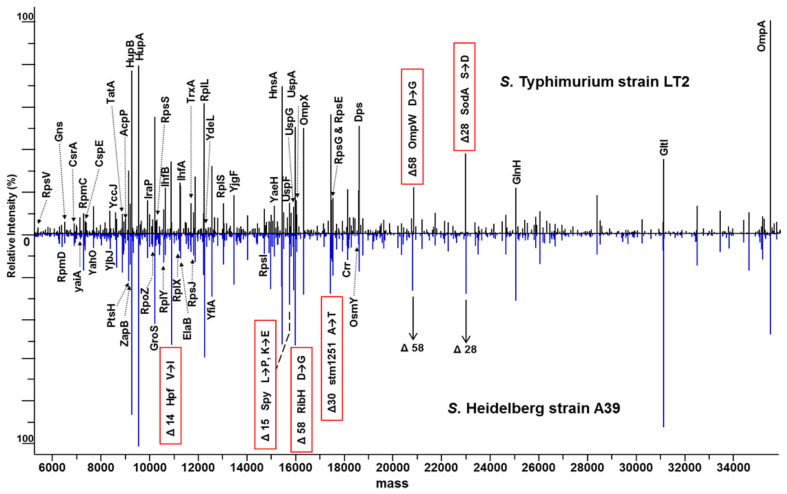
Comparison of intact protein expression profiles for *S. Typhimurium* strain LT2 and *S. Heidelberg* strain A39. A subset of identified proteins is labeled with protein names. Six highlighted masses contain serovar-specific SNP-related mass differences. Amino acid changes are noted from *S. Typhimurium* to *S. Heidelberg* [104]. © American Chemical Society, reprinted with permission.

Initiatives like PathoTOP support the development of clinical applications of TDP with the aim of identifying and characterizing bacterial pathogens. Dupré et al. developed an LC-MS/MS-based TDP platform for *E. coli*, identified 220 proteins and 500 proteoforms, and extended it to analyze enterobacterial pathogens that are not distinguishable using MALDI-TOF-based approaches [107]. There are 170 species in the enterobacterial family, of which 25 account for the majority of clinically relevant strains responsible for different clinical conditions, but are very closely related, making MALDI-TOF-based proteotyping challenging. This study shows that proteoform identification is also database-dependent, and success largely depends on the completeness of the database—a major bottleneck for BUP as well. Neil et al. identified two variants of penicillin-binding protein 2a (PBP2a proteins), PBP2a_mecA_ and PBP2a_mecC_, using a 5-min LC-Top-Down-MS method [108]. McFarland et al. reported 6 marker proteins for identifying 2 closely related *Salmonella serovar* strains, 4 of which were specific to *S. Heidelberg strain* A39 and 2 were specific to *S. Typhimurium* strain LT2 (Figure 5), demonstrating the utility of TDP in differentiating bacterial subspecies that is simpler than genome sequencing, which involves multiple enzymes or PCR targets [104].

Although, they have not reported the amount of proteome they required for TDP analysis, and it is not possible to determine the dry mass of bacteria from the cfu/mL without a standard curve, given that the approximate cell concentration was 8 × 10^10^ cfu/mL, 55% of the total dry mass is proteome content, and their injection volume was 2 µL, if we assume 1 × 10^8^ cfu/mL is equivalent to 1 × 10^−2^ mg/mL of cell dry mass, they injected approximately 8.8 µg into the column for the analysis, which is million times more than the proteome amount derived from a fast growing *E. coli* single cell [109]. TDP also demonstrated utility in differentiating thermal-resistant strains of *Enterobacter sakazakii* [110] and identifying protein biomarkers unique to outbreak-causing strains of *Vibrio parahaemolyticus* [111]. Furthermore, TDP enables the identification of amino acid differences in proteins, which can be compared with genome sequencing data to validate the presence of a novel protein biomarker. Although the benefits of the ESI-based TDP approaches are recognized in the field, the technology needs to be made more accessible and affordable for clinicians and microbiologists for clinical microbiology applications.

### 3.3. Discovery Bottom-Up Proteomics for Antibiotic Resistance Identification

Bottom-up proteomics (BUP) identifies proteins from digested peptides and has demonstrated utility for detecting antibiotic resistance markers [33,34]. Blumenscheit et al. developed an untargeted LC-MS/MS-based proteomics workflow along with a software, rawDIAtect, for detecting both bacterial species and resistance protein markers within 4 h with 98% sensitivity and 100% specificity from a 1.25 µg proteome digest [33]. Cell-derived proteome analysis provides better specificity as the bacterium of interest is cultured for analysis. However, culture-dependent workflows are more time-consuming. Kondori et al. reported a culture-independent method for rapid and accurate identification of *E. coli*, *S. aureus*, and *C. albicans* using BUP, which is 100–1000-fold more sensitive than MALDI-TOF-MS-based proteotyping [112]. On average, a single HeLa proteome is about 200 pg [113], whereas a faster-growing *E. coli* single-cell proteome is 865 fg [109], indicating that to avail the technology stated above, at least a 231-fold increase in sensitivity will be required. This study, however, successfully demonstrated the utility of discovery BUP in identifying resistance-inducing proteins without the need to cultivate bacteria in the presence of different antibiotics. Table 1 lists examples of discovery BUP studies that have successfully identified antibiotic resistance.

Foudraine et al. reported in their study that some resistance-inducing proteins were not detected using LC-MS, although the corresponding genes were present (SHV/LEN in *K. pneumoniae isolates*) or partially present (*bla*_CMY-132_-like genes in *E. coli* isolates and *bla*_DHA_ and *bla*_OXA-9_-like genes in *K. pneumoniae* isolates) in whole-genome sequencing data, reiterating the fact that proteogenomic-based protein annotation could still potentially fail to identify all resistance-inducing proteins [116]. They observed this phenomenon across three antibiotic classes—beta-lactam (meropenem, a 3rd-generation cephalosporin), aminoglycoside (gentamicin, tobramycin), and fluoroquinolone (ciprofloxacin)—in 187 clinical isolates of *E. coli* and *K. pneumoniae*. This study highlights that a single method may not be universal for screening all possible resistances in a clinical specimen. Complementary tools may help improve the clinical management of infectious diseases by enabling more comprehensive screening for resistance.

## 4. Bacterial Single-Cell Proteomics and Antibiotic Resistance Identification

**Figure 6:**
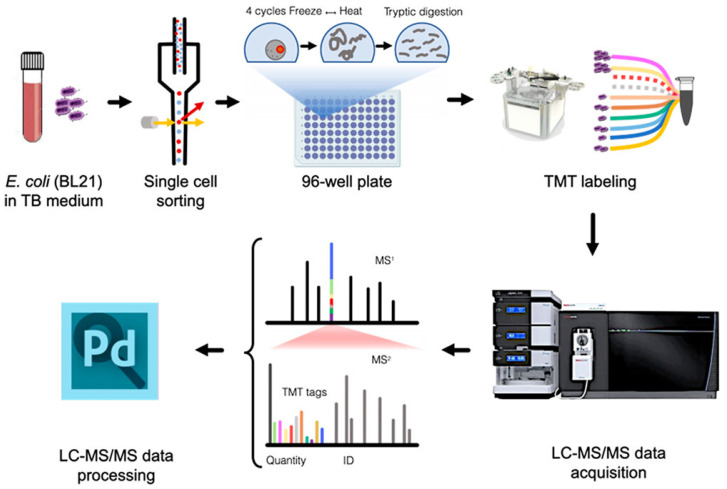
Single bacterium proteomics workflow. The cultured *E. coli* cells were isolated as single or double cells on 96-well plates by FACS; lysed by freeze-and-thaw, and digested before TMT-10plex labeling. Data acquisition was performed on an Orbitrap Fusion Eclipse mass spectrometer equipped with FAIMS Pro, and MS2 spectra were collected for reporter-ion-based quantification [119].

Bacterial single-cell proteomics is a challenging task, primarily because of the extremely small proteome and its dynamic nature, as discussed earlier. To date, only one research article has been published by the Zubarev group that successfully demonstrated the execution of bacterial single-cell proteomics using *E. coli* as a model organism [119]. They used fluorescence-activated cell sorting (FACS) for high-throughput cell isolation and liquid-handling robotics for proteome digestion and peptide labeling, followed by LC-MS/MS-based bottom-up proteomics analysis (Figure 6).

In their pursuit, Végvári and Zubarev used an Orbitrap Fusion Eclipse with ion mobility separation for detecting proteins from single bacterial cells, underscoring the need for advanced instrumentation. They reported identification of 19 proteins from 96 single and double *E. coli* cells, with only three proteins—elongation factor Tu 2, glyceradehyde-3 phosphate dehydrogenase 3A, and 2-aminobutanoate deaminase—each with at least 2 peptide matches. Using a target decoy, they identified 60 proteins, of which 55 had only one peptide match. Thus, it can be realized that the number of detectable proteins from one single bacterial cell in this study represents only a small fraction of the total number of proteins present. For example, Soufi et al. discussed that one single *E. coli* cell contains about 2600 proteins [120], and the number of proteins detected in the above study with at least 2 peptide matches represents only 0.1% of the total protein. Additionally, it should be noted that the resistance-inducing proteins may not be among the most abundant proteins present, which can make it even more challenging to successfully detect resistant protein biomarkers with this approach. To date, no studies have been reported on the identification of resistance-inducing proteins or proteoforms from single bacterial cell analysis. In the following sections, the core challenges of bacterial single-cell proteomics, particularly in detecting antibiotic resistance, are discussed.

## 5. Core Challenges of Bacterial Single-Cell Proteomics

### 5.1. Diversity of Bacterial Populations

The exact number of bacterial species is not known, although it is estimated to range from several million to a trillion [121], of which 30,000 species have been formally discovered [122]. Considering one bacterial species has only one type of cell, there are 30,000 different bacterial cells that differ in size and composition. On the contrary, there are only about 200 distinct cell types in humans [123]. Bartlett et al. reported a total of 1513 human pathogenic bacteria, based on reported infections pre-2021, belonging to 10 phyla and 25 classes [124]. Hence, the number of distinct pathogenic bacteria is approximately 7.5 times higher than the number of different human cell types. Thus, identifying bacteria, sorting individual cells, and conducting proteomics analysis at the single-cell level from a clinical sample for antibiotic resistance detection are extremely difficult [12].

### 5.2. Structure of Bacterial Cell Exterior

Bacteria contain cell walls, an extra layer outside the cell membrane, which is made up of peptidoglycan (PG) crosslinked with lipids that contain several d-amino acids [125]. The presence of the PG layer, unique ribosomes, and DNA gyrase makes antibiotics act only on bacteria, sparing human cells [126]. The PG of the cell wall also provides rigidity while making lysis more challenging. Enzymes such as lysozymes can assist in cell wall damage by breaking glycosidic bonds in the PG layer, but can be less effective against Gram-negative bacteria due to the thickness of their PG layer. Végvári et al. reported measuring 138 more proteins from a pooled proteome digest of *E. coli* using probe sonication compared to freeze–thaw-based approaches, where the former enriched more membrane proteins, while the latter enriched more ribosomal proteins [119]. The success of cellular proteomics relies on the release of the proteome from the cell cytosol, which depends on the cell’s exterior structure and its effective rupture. The approach that works for higher bacterial cellular mass may not work at the single-cell level and may lead to protein loss, rendering bacterial single-cell proteomics analysis unsuccessful.

### 5.3. Bacterial Cell Size and Proteome Amount

A typical human cell is 25 µm in diameter, whereas a single bacterial cell is about 1 µm in diameter; thus, one single human cell can hold more than 10,000 bacterial single cells [127]. To be precise, 15,620 bacterial cells can be accomodated in the volume of a single human cell, assuming both human and bacterial cells are spherical and there is no empty space between them, when comparing volumes based on diameter. This poses a nearly insurmountable challenge for obtaining sufficient proteome for SCP analysis. Recently, Pei et al. developed a technology that can measure proteoforms from single rat microglial cells [17], which typically range from 3 to 6 µm [128]. Calculating volumes based on the cell diameter, this will theoretically reduce the number of single bacterial cells required to measure proteins from 10,000 to 1200 using their technology. Genomic or transcriptomic analyses can circumvent the challenge of limited material by leveraging the PCR-based approach stated earlier, enabling single-cell sequencing, which is not possible with SCP.

The HeLa cell proteome is used as a gold standard for proteomics method development because its proteome is well characterized and contains a large number of proteins [129]. Individual HeLa cells have a diameter greater than 10 µm and a volume of 3000 fL [130,131] with a proteome amount of 150–200 pg [113]. Bacterial cells are several orders of magnitude smaller; hence, sample processing and acquisition workflows optimized for a single HeLa cell may not work for a single bacterial cell. The smallest bacteria, *Mycoplasma gallicepticum*, have a size of 0.2–0.3 μm, whereas the largest bacteria, *Thiomargarita namibiensis*, can be up to 750 μm [132]. The typical diameter of an A549 human cell is 11–15 μm with a volume of ≈1000 μm^3^ [133], while *E. coli* cells represent a cylinder of 1.0–2.0 μm (up to 6 µm) long with a radius of about 0.5–1.5 μm and a volume of ≈1 μm^3^ [134]. Bacteria contain less nucleic acid than eukaryotic cells, and lower nucleic acid content yields a lower proteome per cell [135]. Additionally, the size of a bacterial cell depends on its growth rate; generally, faster-dividing cells are larger than slower-dividing cells. The average dry protein mass of *E. coli* is 148 fg per cell for the strain with a doubling time of 100 min, whereas it is 865 fg per cell (approximately 6.5 times more) for the strain with a doubling time of 24 min [109], which is about 55% of the total dry mass [136]. Comparing the proteome size of a single HeLa (~200 pg) and a faster-growing single *E. coli* cell (~865 fg), to be able to conduct bacterial single-cell proteomics, at least a 231-fold increase in sensitivity would be required. However, for the slower-growing *E. coli* strain (148 fg), a 1351-fold enhanced sensitivity would be required. According to these calculations, one can assume that 200–1300-fold more sensitive technologies than the one enabling single-cell human proteomics would be the ambition to reach to perform bacterial single-cell proteomics and use them to detect antibiotic resistance-inducing protein markers.

### 5.4. Nature and Composition of Bacterial Proteome

Bacterial proteome composition depends on growth conditions, growth rate, and the environment to which they are exposed [137]. Growth conditions, such as nutrient availability, temperature, pH, oxygen levels, and other environmental factors, determine the activation states of cellular pathways, leading to variation in the proteome [138]. Faster-growing cells require increased cellular activity, which in turn requires more ribosomes; hence, their proteome has a higher ribosomal protein content [139]. A difference in the proteome is also reported for *E. coli* when grown in solid vs. liquid culture, as reported by Foutuin et al., who identified 1989 proteins, of which 31% were uniquely expressed in one condition or the other [140]. They also discussed that many proteins associated with virulence and host cell adhesion are often expressed only when grown on solid media, because this mimics biofilm growth. As discussed earlier, many resistance-inducing proteins are only expressed when bacteria encounter antibiotics. The highly dynamic proteome composition of the bacteria makes it challenging to use proteomics for resistance identification. Additionally, challenges associated with abundant proteins in proteomics analysis are well documented, as observed in plasma proteomics, where high levels of albumin, immunoglobulins, and other abundant proteins are present [141]. Structural proteins such as ribosomal proteins constitute a major part of the bacterial proteome and, under normal conditions, may hinder studying proteins that are present in lower amounts. The abundant proteins fill the MS detector and impede the analysis of low-abundant proteins. There are workflows available for depleting abundant proteins, such as antibody-based workflows; however, these workflows require a larger sample volume than can be obtained from a single bacterial cell. Végvári et al. discussed that the proteome of *E. coli* contains 4200 proteins [142], and this number would differ for *E. coli* grown under different conditions and is very different across other bacterial species, further complicating bacterial single-cell proteomics for exploring resistance-inducing proteins.

### 5.5. Identification of Resistance-Inducing Proteins

Not all antibiotic resistance results from gene mutations; hence, identifying protein variants that result solely from genetic mutations may not reliably identify antibiotic resistance. Antibiotic resistance may arise from enzymes that degrade or expel antibiotics or modify the antibiotic targets. Additionally, resistance-yielding proteins may not be present in all strains of a bacterium. Hence, if protein identification is based on a strain lacking the resistant protein sequence in the database, it would go undetected. For example, Erythromycin Resistance Methylase (Erm) is an enzyme that modifies the binding site for macrolide antibiotics, leading to resistance [143], but is not present in all strains of *Streptococcus*, *Staphylococcus*, and *Enterococcus*. However, for their detection, their presence in the database intended for use is mandatory.

Efflux pumps are membrane-associated proteins that remove antibiotics from bacterial cells, making them ineffective [144]. Efflux pumps can be promiscuous and contribute to multidrug resistance, e.g., AcrAB-TolC in *E. coli* [145]. They can also translocate themselves across the cell membrane and into the extracellular space [144], making their detection more challenging. Similar to efflux pumps, porins are also membrane proteins that act as channels for the passive diffusion of antibiotics and small hydrophilic nutrients, as well as filters that provide some degree of control over what enters the periplasmic space [146]. Porins can alter their number and size to restrict the entry of antibiotics into the cell, leading to a gradual and slow exposure to antibiotics, which results in antibiotic resistance [147]. Proteomics analysis of bacterial porins [148] and efflux proteins [149] from liquid culture is reported via bottom-up proteomics; however, achieving such performance from a single bacterial cell does not seem feasible at this moment.

Resistance-inducing proteins may also vary in sequence and size among different bacterial species due to continuous evolutionary pressure, horizontal gene transfer across bacterial species and subspecies, and vertical gene transfer during cell division [150], complicating their identification. Additionally, as discussed before, resistance-inducing proteins may not be among the most abundant proteins in the bacterial proteome, as resistance development depends not only on protein quantity but also on its stability, catalytic efficiency, binding affinity, and other factors [151]. In fact, bacteria are known to employ several strategies that do not require abundant proteins to combat antibiotics, rendering them ineffective [152]. These challenges make the identification of resistance-inducing proteins more complex.

### 5.6. Data Analysis

**Figure 7:**
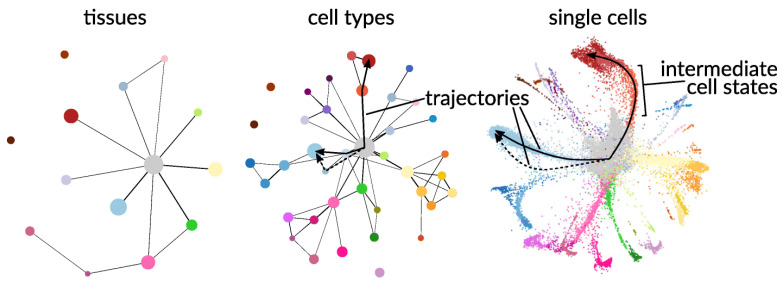
Partition-based graph abstraction (PAGA) for RNA-seq data for the flatworm, *Schmidtea mediterranea*, at tissue, cell type, and single-cell resolution [153]. Each color represents distinct cell clusters or groups based on transcriptional composition.

Single-cell experiments generate large, high-resolution datasets that must be handled in a computationally and statistically efficient manner. Figure 7 illustrates the high level of heterogeneity that exists at the single-cell level, where the single-cell tree is built from single-cell RNA-seq data from 21,612 single cells of the flatworm, *Schmidtea mediterranea*.

The limited amount of proteome from bacterial single cells can also lead to uncertainty about the observations. Lähnemann et al. [153] discussed the grand challenges of single-cell data science for genomics; however, most of these challenges are correct for proteomics as well, such as missing values, flexible statistical analysis to detect unique features, integration of data across samples, experiments, and types of measurements, as well as validating and benchmarking analytical tools for single-cell measurement. The very low proteome derived from a single bacterial cell is reported to yield a high number of missing values [154]. Additionally, due to the large number of bacterial species, it is difficult to standardize a universal computational workflow for SCP analysis. Table 2 lists the primary data analysis challenges in single-cell proteomics.

Single-cell methods generate high-dimensional, large datasets, computation of which is complex and burdensome [160]. Integration of additional omics datasets increases the complexity of managing and analyzing this multidimensional dataset. Correlating different omics data is another challenge that requires proper planning before data acquisition. Successful integrative omics analysis also depends heavily on effective communication among biologists, analytical scientists, and bioinformaticians. Analysis of complex multidimensional SCP data will greatly benefit from machine learning and artificial intelligence, particularly for data imputation, cross-experiment matching, and correlative data modeling [161,162].

## 6. Emerging Technologies for Bacterial Single-Cell Proteomics

Treatment of infectious diseases caused by pathogenic bacteria has benefited from high-throughput workflows through species identification, antibiotic susceptibility testing [163], and drug screening [41,164,165,166,167]. Resistance-inducing protein identification using proteomics at the single-cell level would also require high-throughput workflows for extracting, processing, and analyzing proteomes from hundreds or thousands of bacterial single cells. High-throughput antibiotic screening workflows are based on optical detection; however, detection and/or quantitation of molecules (e.g., proteins) responsible for inducing antibiotic resistance via mass spectrometry may require additional handling and labeling steps [168], emphasizing the need for high-throughput workflows. Modern microfluidics-based technologies enabled single-cell proteomics analysis, but were built around the gold standard HeLa or other mammalian cells [169,170]. However, due to the significantly lower proteome content of a single bacterial cell compared to a single mammalian cell, and the fact that hundreds or thousands of single cells would require analysis, high-throughput workflows are essential and necessitate miniaturization with fewer steps.

Methods that use labeling, such as fluorescence-activated cell sorting (FACS), as well as label-free technologies, such as cellenONE, are capable of isolating single bacterial cells in microliter volumes in a high-throughput fashion, and the latter demonstrated isolating 96 single bacterial cells in 3 min from a liquid culture [171,172]. Single-cell ink-jet printer demonstrated success in automated isolation and deposition of single bacterial cells in 35 pL droplets [173]. Lab-on-a-chip is a miniaturized device that enables isolation and entrapment of single bacterial cells in a small volume (pL to nL) of liquid (microfluidic droplet or nanowell), followed by incubation and buffer exchange [174,175,176] that can potentially be interfaced with mass spectrometers. Miniaturized flow reactors also allow the incubation of cells under different environmental conditions, facilitating the cultivation of bacteria that require specific conditions, such as temperature and pH [172], or were previously difficult to grow by mimicking their natural habitats [177]. Cell lysis on a lab-on-a-chip device can be achieved via electroporation, which creates pores in the bacterial membrane, releasing cytosolic materials, including proteins [178]. Peindl et al. demonstrated the use of a microfluidic device that can transfer nL-sized droplets into a chip nano-LC system for MS analysis [179]. Liu et al. developed a polymeric microchip integrated with solid-phase extraction via a monolithic trap column, enabling online cleanup and reverse-phase HPLC analysis of 11 ng of peptides (tryptic digests of BSA) and 57 ng of proteins (ribonuclease A and cytochrome C) [180]. Direct hyphenation of these systems from cell sorting, proteome extraction, and processing to mass spectrometry analysis is yet to be seen.

BUP requires additional sample preparation steps, such as denaturation, reduction, alkylation, and enzymatic digestion prior to injection into the mass spectrometer [181]. And technologies to perform these steps in miniaturized formats are commercially available, providing a true walkaway setup [182], as well as semiautomated solutions using custom-made devices and software that offer more control over injection [182]. Among many novel technologies, the Chip-Tip workflow demonstrated the best results to date, identifying more than 5000 proteins from a single HeLa cell, including direct PTM detection, and achieving a throughput of 120 label-free samples per day [169]. A similar automated BUP workflow for bacterial proteomics was also reported, which can extract proteins from bacterial cell pellets and digest 96 samples in a microwell plate within 2 h, with the capability to parallel-process 384 samples [183]. Due to the requirement of screening a large number of single cells, peptide labeling agents such as TMT, iTRAQ, mTRAQ, SILAC, etc., can provide multiplexing ability, increasing throughput; however, they limit the ability to operate the analysis in a dynamic mode where new samples cannot be added to the existing run [184]. When dealing with such a low proteome amount, a slight loss of sample due to adsorption to the surface of consumables can lead to failure; hence, workflows with minimal sample transfer and that essentially conduct all steps in a single chamber could be highly beneficial [185]. Data acquisition technologies, such as data-independent acquisitions (DIA) with expansive isolation windows, are believed to provide greater sensitivity to low sample input and could be explored for bacterial single-cell proteomics; however, coupling DIA to peptide labeling needs to be carefully evaluated [186]. Data-dependent acquisition (DDA) with wide-window acquisition and ion mobility spectrometry are other avenues that could be explored for analyzing single-cell proteomes [22] and could help attain the analytical performance required for bacterial single-cell proteomics.

Besides miniaturizing bacterial cultivation and sample processing, miniaturization of ionization and ion transmission to a mass spectrometer via a sheath–liquid interface in capillary electrophoresis (CE)-ESI-MS also demonstrated an increase in signal for the tryptic digest of BSA [187]. Such an interface would be better suited for a lab-on-a-chip-derived microfluidic droplet due to reduced dilution effect and higher surface-to-volume ratios. Capillary electrophoresis mass spectrometry (CE-MS) could be further explored, especially to achieve higher throughput and user-friendliness, given its ability to analyze the proteome at injection volumes ranging from pL to nL [188], a volume regime that a single bacterial cell’s proteome falls within. Moreover, technological capabilities to perform various steps of bacterial single-cell proteomics in a high-throughput fashion exist, and we await seeing the next revolution in this field, i.e., determining antibiotic-resistant single bacterial cells via proteomics.

## 7. Beyond Bacterial Cellular Proteomes for Antibiotic Resistance Identification

Antibiotic resistance is a naturally occurring phenomenon that cannot be entirely eradicated due to the unavoidable selective pressure of antibiotics [189]. However, it can be managed through antibacterial stewardship, appropriate preparedness, and the availability of new antibiotics, all of which require sustained effort and collaboration among healthcare professionals, policymakers, researchers, pharmaceutical companies, and world leaders. Detecting antibiotic resistance through molecular methods and assessing susceptibility using routinely used phenotypic methods are the stepping stones to effectively managing bacterial infections. Proteomics is a powerful technique that can aid in managing infectious diseases if used appropriately to its strengths. Although using cellular proteomics as a tool for resistance determination at single-cell resolution is a complex pursuit, with clinical success yet to be fully realized, studying bacterial secretory, membrane, and biofilm proteomes can further deepen our understanding of the mechanisms of antibiotic resistance development, as discussed in this section.

### 7.1. Bacterial Secretory and Membrane Proteome for Antibiotic Resistance Identification

The role of bacterial secretory proteins in inactivating antibiotics and membrane proteins expelling antibiotics, leading to antibiotic resistance development, is commonly observed against beta-lactam antibiotics [190], a major antibiotic resistance challenge [191]. Additionally, bacterial membrane and secretory proteins play a major role in safeguarding bacteria against adverse conditions by facilitating nutrient uptake, cell adhesion, biofilm formation, cellular communication via virulence factors, and the functional dysregulation of host cells [192]. Secretory proteins are transported via secretion machineries residing on the cell membrane, which differ in structure, function, and the proteins they carry, and can be used as antibiotic targets [193]. Secretion apparatus, like type 4 secretion system (T4SS), is capable of transporting proteins and DNA across membranes in bacteria and into the host cells, and plays essential roles in the pathogenesis of a wide range of bacteria, e.g., *Neisseria gonorrhoeae* (virulence gene acquisition) [194], *Legionella pneumophila*, *Brucella suis*, and *Helicobacter pylori* (disrupt the host’s defense repertoire) [195] and spread resistance genes via horizontal gene transfer. Other secretory proteins or assemblies, such as aSec sortases, pilin, and the injectosome, contribute to pathogenesis via pore formation and transportation of virulence factors, such as *Streptococcus pyogenes* NAD glycohydrolase (SPN) [195,196]. Bacteria with an additional membrane, such as mycobacteria, use the T7SS, which participates in virulence regulation [197]. Secretory proteins are also reported to sequester antibiotics, forming complexes, preventing their entry into bacterial cells, or preventing their binding to the target, as seen with bleomycin antibiotics, which bind to proteins TlmA, BlmA, and ZbmA, and mitomycin, which binds to the MRD protein [198]. Hence, examining the bacterial secretory proteome and the secretory assemblies, such as the membrane proteome, may help to look beyond host–pathogen interactions and identify novel resistance-inducing proteins or mechanisms for resistance development. Mass spectrometry-based proteomics has been successfully used to study bacterial outer membrane proteins [199], secretory proteins, and their substrates [200]. However, the secretome composition is dependent on the environment in which bacteria reside and would differ with changes in their growth environment, e.g., lab-grown environment vs. host biomaterials (cell, blood, etc.), and also during antibiotic exposure [201]; hence, they need to be carefully considered for resistance protein identification via proteomics.

Antibiotics that interact with intracellular targets, e.g., DNA or RNA biosynthesis inhibitors, must be transported into the cell to reach their target sites. Resistant bacteria may reduce the expression of membrane transport proteins, lowering the entry of antibiotics into the cell and impeding the achievement of the therapeutic concentration [152]. Identifying and quantifying such proteins in a resistant bacterial population can help understand mechanisms of resistance development, the causes of therapeutic failure, and identify resistance markers. A major group of bacterial transmembrane proteins is efflux pumps that maintain bacterial cellular homeostasis and transport various molecules across the cell membrane [202]. Efflux pumps can expel one or many classes of antibiotics from the cell, contributing to antibacterial inefficacy as observed with ATP-binding cassette (ABC) transporters [203]. Efflux pumps have also been reported to participate in biofilm formation [204], which can lead to antibiotic resistance, as discussed in the next section. Efflux pumps play a role in regulating bacterial virulence, pathogenicity, and host response, contributing to antibiotic resistance during infection [205]. Overexpression of efflux pumps has also been reported to be associated with antibiotic resistance [206] and can serve as markers for such. Six major classes of efflux pumps have been identified in bacteria, such as the ATP-binding cassette (ABC) superfamily, the major facilitator superfamily (MFS), the multidrug and toxic compound extrusion (MATE) family, the resistance nodulation cell division (RND) family, the small multidrug resistance (SMR) family, and the proteobacterial antimicrobial compound efflux (PACE) family [205]. One type of efflux pump can transport many classes of antibiotics, and one class of antibiotics can be transported by more than one type of efflux pump, making them a greater contributor to antibiotic resistance and explaining why developing their inhibitors is a tremendous challenge [144]. Several efflux pump proteins from the RND and MFS families have been reported to be involved in developing antibiotic resistance, including AcrAB-TolC in *E. coli* and other *Enterobacteriaceae*; MexAB-OprM, MexXY-OprM, MexCD-OprJ, and MexEF-OprN in *P. aeruginosa*; AdeABC in *Acinetobacter*; Tet38, NorA, NorB, and NorC in *S. aureus*, etc. [207,208,209,210,211]. Besides efflux pumps, bacteria also have passive protein channels known as porins that facilitate the influx of small molecules, nutrients, and antibiotics [212]; alterations in their structure and reductions in their number can also lead to antibiotic resistance [147]. The bacterial outer membrane proteome undergoes remodeling, and the types of proteins expressed differ depending on growth conditions [212]. Proteomics analysis has demonstrated success in identifying porins [213]; for example, reduced porin expression was reported in carbapenem-resistant *E. cloacae* [213]. Although proteomics studies of bacterial membrane proteins have been reported [199,214], their isolation is relatively more challenging than that of intracellular or secretory proteins, due to their transmembrane anchoring, propensity for complex formation, hydrophobic nature, and association with a complex lipid environment [215]. Additionally, distinguishing porins can be challenging; for example, OmpC and OmpF are structurally almost identical and exhibit very limited sequence variation [216]. Further advancements in membrane protein enrichment and analysis will enable a deeper understanding of their role in the development of antibiotic resistance and greatly aid in identifying multidrug resistance.

### 7.2. Biofilm Proteome for Antibiotic Resistance Identification

Bacterial biofilm consists of water, cells, and cell-derived substances, including polysaccharides, proteins, lipids, and nucleic acids [217]. It protects bacteria against adverse environments, such as antibiotic exposure, and helps recycle nutrients to help with survival in nutrient-deficient conditions [218]. Bacteria that can produce biofilms demonstrate significantly reduced susceptibility to antibiotics compared to non-biofilm-producing bacteria [219]. Biofilms produced by different bacteria differ in their proteome compositions and functions [220] and respond differently to various antibiotics and their doses, resulting in antibiotic resistance that leads to therapeutic failure [220]. The biofilm proteome differs significantly from the cellular proteome and is primarily composed of secretory proteins, outer membrane vesicles, cell-surface adhesion proteins, as well as proteins derived from pili and flagella [221]. Besides the storage and exchange of genetic materials among bacterial cell populations [222], biofilm provides an adaptive antibiotic resistance mechanism, which is unrelated to genetic mutations [218]. Proteomics has been successfully used to study the biofilm proteome [223,224,225] and its link to quorum sensing, cellular communication, stress response, and energy metabolism [226,227].

Antibiotics that are positively charged, e.g., streptomycin and gentamicin, encounter difficulties entering the biofilm, leading to uneven distribution, enabling bacteria to adapt and survive upon further antibiotics’ exposure making them ineffective [228]. Besides resisting the entry of antibiotics, biofilms can also trap, modify, degrade, or inactivate them, thereby rendering them ineffective [229,230]. Biofilms develop gradients of nutrients, oxygen, pH, hydrogen peroxide, and metabolites, in which the bacteria in the central part of the biofilm have a reduced capacity to grow compared to those on the biofilm surface, a phenomenon that also occurs with antibiotics, leading to the development of antibiotic resistance [218]. Many biofilm proteins have already been identified as markers for antibiotic resistance, e.g., curli fimbriae, elongation factor G against aminoglycosides; ribosomal protection proteins, Las(A), Msr(A), Optr(A), and Vga(A) against macrolide, streptogramins, and phenicols; (p)ppGpp regulatory proteins, etc. [231,232,233,234]. However, it should be kept in mind that biofilm-induced resistance is complex and multifactorial, as it is dynamic and varies across species, strains, locations, time, and environment. Hence, identifying biofilm proteins as markers of antibiotic resistance can be challenging, and integrating them with other omics studies, such as metabolomics, transcriptomics, and genomics, can enhance their use in understanding resistance development.

## 8. Future Outlook

Unlike nucleic acids, proteins cannot be amplified in their natural form as they lack the template for multiplication [235]. Indirect methods like AmproCode, although demonstrated the capability to increase the relative measurement of proteins by using amino acid labeling via DNA barcodes, have labeling efficiency that varies with amino acids and inaccuracy at the proteome level in complex environments [236]. Direct methods, such as nanopore-based protein sequencing, can analyze a protein’s amino acid sequence by measuring changes in ionic current as it passes through a tiny nanopore [237], a promising technology for identifying disease-associated protein biomarkers. Lu and Maglia discussed the likelihood of nanopores’ capability to analyze full-length proteins at the single-molecule level and with single amino acid resolution that may enable measuring PTM-driven cellular heterogeneity, quantification of low-abundant proteins, and characterization of protein splicing [238], all of which make it a promising technology for antibiotic resistance detection, similar to nanopore-based DNA or RNA sequencing [239]. A more reasonable alternative to bacterial single-cell proteomics may be single-colony proteomics, in which all visually distinguishable colonies from a specimen undergo proteomics analysis after identification of the bacterial species. However, such a workflow will still take longer than a day, which is similar to or longer than the duration that culture-based methods routinely take (typically 18 to 24 h) in identifying bacteria and testing their antibacterial susceptibility [240].

The therapeutic goal of resistance identification is to find an antibiotic that can eradicate pathogenic bacteria and stop the spread of the infection, i.e., to determine the antibiotic susceptibility of the causative pathogen. Although understanding the mechanism of resistance development is important, from a clinical perspective, treating the infection caused by the pathogen is more critical. Hence, emerging technologies are focused on developing highly sensitive, accurate, and fast antibiotic susceptibility testing workflows and aim to utilize next-generation technologies, including bacterial single-cell imaging, plasmonic bacterial sensing, deep learning, and highly multiplexed nucleic acid probing [241]. Single-cell antibiotic susceptibility testing (SC-AST) that tracks phenotypic changes via microscopy and deep learning demonstrated success in distinguishing susceptible vs. resistant single bacterial cells when treated with different antibiotic classes such as the fluoroquinolone ciprofloxacin (which targets DNA synthesis), the aminoglycoside gentamicin (targets protein synthesis), the beta-lactam co-amoxiclav (targets cell-well synthesis), and rifampicin (targets RNA synthesis) within 30 min [240]. Microfluidics also demonstrated success in high-throughput single-cell AST for both Gram-positive and Gram-negative bacteria, based on the size of the single cell, determining phenotypic heterogeneity [242], bacterial identification, and antibiotic susceptibility from urine, blood culture, and whole blood in 30 min [243]. Single-cell morphological analysis (SCMA), integrating microfluidics and microscopy, demonstrated success in determining antibiotic susceptibility by automatically analyzing and categorizing morphological changes in single bacterial cells when exposed to antibiotics within 4 h [244].

In 2019, Welker and Belkum discussed the unlikelihood of mass spectrometry as an alternative for conventional antibiotic susceptibility testing in the future [57]. However, they expressed that the use of mass spectrometry for routine microbial identification also seemed unlikely 15 years before it became a preferred procedure in clinical settings. Although proteomics has aided in understanding bacterial resistance mechanisms by identifying proteins’ quantity, PTMs (e.g., acetylation, oxidation, formylation, phosphorylation) [99,245], turnover rates, and subcellular location [59]; to fully utilize the potential of mass spectrometry for antibiotic resistance testing, it is necessary to go beyond measuring the proteome to study the metabolome, lipidome, and glycome [246]; to monitor antibiotic degradation, co-expressed biomarkers, and bacterial growth [57,247]. The genome directs what needs to happen, which the transcriptome carries to the proteins that execute the function with the help of metabolites. Metabolites are effector molecules that are involved in all essential physiological pathways, such as cell wall, nucleic acid, protein, amino acid biosynthesis, as well as carbohydrate, peptidoglycan, fatty acid, lipopolysaccharide, and lipid metabolism, and are studied comprehensively in response to antibiotic treatment [248,249]. Advanced instrumentation and bioinformatic workflows have enabled large-scale integrative omics analysis for novel metabolite discovery [250,251,252] and can significantly help in profiling bacterial metabolome for resistance-inducing biomarker discovery. Proteomics and metabolomics sample preparation can vary due to the chemical diversity of the metabolome [253]; therefore, they should be critically assessed. Innovative technologies, such as the Microbial Containment Device (MCD) developed by Mohammadi and Lewis, analyze water-soluble metabolites in the culture medium without extraction to study the effect of antibiotic perturbation, making them noteworthy [254]. Additionally, integrative omics studies generate multidimensional datasets, which can be challenging to analyze. Artificial intelligence and machine learning models have the potential to streamline data processing and management and to aid in deriving meaningful biological insights from complex multidimensional datasets [255]. Microdroplet batch reactors offer a high degree of parallelization and could potentially be coupled to MS-based workflows for single-cell omics studies [256]. The number of bacterial single-cell proteomics and metabolomics studies is limited, and most published studies focus on innovation and feasibility, lacking the rigor of bench-to-bedside translation. Promising technologies for single-cell omics analysis have been developed over the last decade, and their clinical application testing will benefit from collaboration between academia and industry.

## 9. Conclusions

The two major cornerstones of clinical microbiology are to determine (1) the identity of pathogenic microorganisms and (2) their antibiotic susceptibility and resistance profile. Proteomics has revolutionized bacterial identification in clinical settings and has also demonstrated success in identifying proteins responsible for the development of antibiotic resistance, as discussed earlier. Technological advancements enabled the identification of bacteria from single-cell analysis via MALDI-aerosol time-of-flight mass spectrometry [257]. Although antibiotic resistance development is primarily regulated by proteins, they may not always be the only drivers of resistance development [258]. Since protein identification in proteomics analysis depends on gene expression and sequence data, determining antibiotic-resistance marker proteins through proteomics is not possible without prior knowledge of the bacterial species. Hence, it may not be reasonable to use proteomics as a single, universal technique for determining antibiotic resistance in a clinical setting, as it would require screening a large database of every known pathogenic bacterium and all of its antibiotic resistance-inducing proteins. Databases such as “The Comprehensive Antibiotic Resistance Database (CARD)” (https://card.mcmaster.ca/, accessed on 26 October 2025) [259], “NCBI National Database of Antibiotic Resistant Organisms (NDARO)” (accessed on 26 October 2025) [260], and other similar databases [261] can significantly aid in identifying antibiotic resistance markers, and they require constant updating and monitoring. Bacterial single-cell proteomics is more challenging due to the extremely low proteome content, the isolation and measurement of these cells, especially for resistance-inducing identification, as they may not always be among the abundant proteins [262], as discussed earlier. Additionally, there are other mechanisms through which resistance develops, e.g., enzyme production by bacteria that modify the antibiotics (e.g., acetylation, phosphorylation, or adenylation of antibiotics) in the periplasmic space, reducing drug permeability through modifications of lipopolysaccharide, membrane composition, decreasing proton motive force, as well as increasing drug efflux, as can be seen for resistance against aminoglycosides [68]. Besides screening for known resistance marker proteins, mass spectrometry can greatly aid in studying resistance induced by these other mechanisms, such as detecting antibiotic degradation products, modified lipopolysaccharide, or protein–antibiotic interactions, indicating the synergy that integrative omics studies can bring.

Given the current state of the field, it is not feasible to explore bacterial single-cell proteomics for the identification of antibiotic resistance. MADLI-TOF-based proteotyping should be continually updated to include new bacterial species, aiming to achieve a more comprehensive classification at the subspecies and strain levels. Furthermore, continued proteomics analysis of newly identified pathogenic and non-pathogenic bacteria upon antibiotic exposure and their integration into existing databases will help identify protein biomarkers of antibiotic resistance. These efforts will ensure the availability of a more reliable and clinically relevant database for resistance identification. Emerging technologies, including single-cell sorting, microfluidic-based workflows, lab-on-a-chip, and automated liquid handlers, have enabled high-throughput bacterial cultivation and sample preparation. Future long-term endeavors should focus on assessing the ease of their integration into clinical labs and disseminating training on these technologies, which may pave the way for the use of bacterial single-cell proteomics in antibiotic resistance detection.

Antibiotic resistance testing identifies which antibiotics will not be effective in treating infections, whereas antibiotic susceptibility testing identifies the ones that will be effective. While the former is important for understanding why resistance develops and perhaps for preventing future resistance, the latter is more crucial from a clinical perspective for determining the effective treatment plan against the infection. Emerging technologies in sample preparation, data acquisition, hardware configuration, data analytics, and deep learning may open new doors for determining antibiotic resistance and susceptibility through mass spectrometry-based workflows. It remains to be seen how those workflows contribute to bacterial single-cell proteomics in addressing the antibiotic resistance-driven global health crisis.

## Figures and Tables

**Table 1:** List of discovery bottom-up proteomics studies identifying antibiotic resistance ^a^.

Tested Antibiotic/Antibiotic Class	Bacterial Species	Sample Type	Marker Proteins/ Peptides/Genes	Quantification	Additional Test	Ref.
Ceftriaxone Gentamicin Carbapenem	*A. baumannii* (3 isolates)	Respiratory tract, blood, and urine samples from hospitalized patients	I6TUC8, Q0GA83 O05286, D0CCK1, Q2FCY1 L9LWL7, L9MDB0, K9C9W3, E2IGU7, B6E129, G8HYR7, D2XTB0,D2XTB0	iTRAQ (isobaric tags for relative and absolute quantification)	Gene ontology functional enrichment analysis	[114]
Cefotaxime	*E. coli*, *P. aeruginosa*, *S. aureus*, *S. pneumoniae*, *M. catarrhalis*, *H. influenzae*	Respiratory tract and urine samples of an infected 2-year-old boy	CTX-M,	Not performed	In silico analysis	[115]
			2. β-lactamase *bla^TEM−1^*, 3. *aac*(*3*)*-II*			

Beta-lactams Aminoglycosides Fluroquinolones	*E. coli* (78 clinical isolates) *K. pneumoniae* (109 clinical isolates)	Stored clinical isolates	^b^1a. Carbapenemases (NDM, OXA-48, KPC, VIM) ^b^1b. Beta-lactamases CTX-M, TEM, OXA-1	Label-free quantification	Susceptibility testing, WGS	[116]
			^b^2. 16S-RMTases ^b^ (*armA*, *rmtB*, *rmtC*, *rmtF*, *RmtB*) ^b^3. QnrA, QnrB, AAC(6′)-Ib-cr, *oqxA*, *oqxB*			
Beta-lactams Tetracycline	Tested against different bacterial phyla (see reference for complete list)	Raw cow milk	^b^TET, LRA-19, IND-16, OXA-658, MPHN, TET (52), NIMC, BRO-2, CFRC	Label-free quantification	LAP-MALDI, metaproteomics	[117]
Beta-lactams Streptomycin Macrolide Aminoglycosides Sulfonamide Polymyxin	*E. coli*, *K. pneumoniae*, *A. baumannii*, *P. aeruginosa*, *S. enterica*	Blood, urine, stool, respiratory tract, and tissue samples	^c^16S rRNA methyltransferase, O-phosphotransferase, N-acetyltransferase, Macrolide phosphotransferase, Nucleotidyltransferase, Beta-lactamase, PBP1b, MFS, RND efflux pumps, Sulfonamide resistant sul, Bifunctional polymyxin resistance protein (*arnA*)	Label-free quantification	Genomic analysis	[118]

^a^ This is not an exhaustive list. ^b^ Full list of resistance genes can be found in Foudraine et al. [116]. ^c^ 16S-RMTases: 16S ribosomal RNA methyltransferases, an aminoglycoside-modifying enzyme (AME). LAP-MALDI: Liquid atmospheric pressure matrix-assisted laser desorption/ionization. AAC: Aminoglycoside acetyltransferase. Light Blue Shading: UniProt Accession Number. Light Green Shading: UniProt Gene Name. Light Orange Shading: Protein/Enzyme Name.

**Table 2:** Key challenges of SCP data analysis.

Key Challenge	Key Consideration	Ref.
Data Normalization Inappropriate normalization or a lack of normalization can lead to comparing technically variable data and result in an inaccurate comparison of protein abundances between cells	To address differences in protein abundances among single cells, the use of a common subset of proteins or a standard reference should be considered	[155,156]
Missing Values Depends on MS acquisition settings (e.g., random for TopN DDA vs. low abundance for DIA)	Comprehensive imputation methods for SCP are not available, and assessing different methods is recommended	[157]
Reproducibility Due to the uniqueness of individual cells, a common feature is needed to assess reproducibility	Internal standard Cell clusters	[157]
Dimensionality Use of a mismatched application for high-dimensional SCP data may lead to exclusion of important biological variability	Robust statistical design Orthogonal validation, e.g., SingPro, which enabled comparing MS-based SCP to flow cytometry-based SCP	[157,158]
Quantitative accuracy Abundant features vary between bulk and SCP Many different features may represent biological and time-induced proteomic variability among single cells, and their accurate quantification may require dedicated acquisition methods	Statistical power to differentiate single cells based on significant feature variability Targeted analysis post discovery analysis with authentic and internal standard Signal booster agents such as tandem mass tag	[159]

## Data Availability

No new data were created or analyzed in this study. Data sharing is not applicable to this article.
